# Intra- and Inter-Rater Reliability of Primitive Reflex Assessments in Youth Football Athletes

**DOI:** 10.3390/jfmk11030324

**Published:** 2026-08-21

**Authors:** Julie Bastiere, Aurélien Patoz, Thibault Lussiana, Marvin Subryan, Cyrille Gindre, Laurent Mourot

**Affiliations:** 1 SINERGIES, Université Marie et Louis Pasteur, 25000 Besançon, France; 2 Plateforme Exercice Performance Santé Innovation, Université Marie et Louis Pasteur, 25000 Besançon, France; 3 Research and Development Department, ESTAC Association, 10000 Troyes, France; 4 Research and Development Department, Volodalen, 1023 Crissier, Switzerland; 5 Institute of Sport Sciences, Biomechanics & Integrative Physiology of Exercise Dynamics (BIPED) Laboratory, University of Lausanne, 1015 Lausanne, Switzerland; 6 Research and Development Department, Volodalen, 39270 Chavéria, France; 7 Department of Biological Science, Thompson Rivers University, Kamloops, BC V2C 0C8, Canada

**Keywords:** Institute for Neuro-Physiological Psychology, psychometric properties, measurement reproducibility, evaluator expertise

## Abstract

**Background**: Primitive reflex (PR) assessments based on the Institute for Neuro-Physiological Psychology (INPP) framework are increasingly used in developmental and athletic populations. However, the reproducibility of these observer-based assessments remains insufficiently documented. This study investigated the intra- and inter-rater reliability of subjective PR assessments and examined the potential influence of evaluator expertise in elite youth football athletes. **Methods**: Sixty-nine male youth football players from an elite academy (age: 18 ± 2 years) participated in this study. PR assessments included the Moro reflex, asymmetrical tonic neck reflex, symmetrical tonic neck reflex, and tonic labyrinthine reflex, evaluated using standardized INPP procedures (0–4 ordinal scoring scale). Intra-rater reliability was assessed by an experienced examiner across three testing sessions performed over six months. Inter-rater reliability was evaluated using standardized video recordings independently scored by two novice raters. Relative reliability was assessed using intraclass correlation coefficients (ICCs) with corresponding 95% confidence intervals (95% CIs), while absolute reliability was quantified using the standard error of measurement (SEM) and the minimal detectable change at the 95% confidence level (MDC95). **Results**: *Good* to *excellent* intra-rater relative reliability (ICC = 0.86–0.98; 95% CI = [0.81–0.99]) was observed, with SEM values of 0.1–0.6 and MDC95 values of 0.3–1.7 points. *Moderate* to *excellent* video-based inter-rater reliability was found (ICC = 0.69–0.99; 95% CI [0.56–0.99]), with SEM values of 0.1–0.7 and MDC95 values of 0.3–2.0 points. Pairwise comparisons among raters showed *moderate* to *excellent* relative reliability (ICC = 0.57–0.99; 95% CI [0.35–0.99]), with SEM values of 0.1–0.5 and MDC95 values of 0.2–1.3 points. **Conclusions**: Subjective PR assessments performed using the INPP framework demonstrated *good* to *excellent* intra-rater relative reliability and *moderate* to *excellent* video-based inter-rater relative reliability, together with generally low absolute measurement error in elite youth football athletes. These findings provide preliminary psychometric support for the use of standardized PR assessments in research and applied sport settings.

## 1. Introduction

Primitive reflexes (PRs) are automatic motor responses emerging during fetal and early neonatal development and primarily mediated by subcortical and brainstem networks [1,2,3]. During typical neurodevelopment, these reflexes are progressively inhibited as supraspinal motor control matures, contributing to the emergence of postural regulation and voluntary movement [4,5]. However, this developmental inhibition should not necessarily be considered an all-or-none process, as observable PR activity may persist beyond childhood or re-emerge later in life. Because marked persistence or re-emergence of PRs has historically been associated with altered neurological functioning, their assessment has traditionally been incorporated into developmental neurological examination and pediatric clinical practice [6,7].

Beyond neonatal neurology, PR activity has been reported across different stages of the lifespan and in a variety of populations. Persistent PR activity has been described in children and adolescents [8,9,10,11], while associations between retained PRs and cognitive function have also been reported in specific populations of adolescents and adults [12]. PR activity has additionally been documented in adults with sensorimotor disorders [13] and may re-emerge or become more evident with advancing age [14,15]. Direct evidence specifically characterizing PR persistence in neurologically healthy young adults remains comparatively limited. Nevertheless, the available findings indicate that observable PR activity is not exclusively restricted to infancy and may vary across individuals and across the lifespan. Some investigations have further identified associations between PR activity and motor coordination, balance, attentional processes, learning-related outcomes, or cognitive function [16,17,18]. However, these findings remain heterogeneous and should be interpreted cautiously due to substantial variability in conceptual frameworks, operational definitions, populations, and assessment procedures across studies.

Interest in PR activity has more recently extended to non-clinical athletic populations. A case study conducted in two 7-year-old children suggested that higher levels of active PR may be associated with longer acquisition times and lower technical proficiency during swimming skill learning [19]. More recently, PR activity has been reported in a large proportion of elite youth football players [20] and has been associated with reduced performance in situations characterized by high spatio-temporal constraints, such as duels and actions performed under pressure [21]. Young athletes therefore represent a particularly relevant population in which to investigate PR activity because they combine a non-clinical status with extensive exposure to training and to motor tasks requiring high levels of coordination, postural control, and rapid adaptation to environmental constraints. In football specifically, these capacities are continuously challenged by rapidly changing interpersonal and environmental constraints, requiring players to perceive, adapt, and reorganize movement within very short time frames. Accordingly, if PR activity reflects meaningful inter-individual differences in sensorimotor organization, its assessment could potentially contribute to the characterization of individual motor profiles and to a better understanding of variability in performance under highly constrained situations. However, such potential sporting relevance should not be interpreted as evidence that PR activity directly determines performance, nor does current evidence support its use for talent identification or clinical screening. Rather, the observation of inter-individual differences in PR activity despite a high level of motor practice raises the question of whether these responses may provide complementary information about sensorimotor organization in trained populations. Within a constraint-based framework, observable PR activity could therefore be considered a potential behavioral marker of variability in motor organization, particularly under conditions requiring rapid adaptation to increasing coordinative and environmental demands [22,23]. Before such observations can be meaningfully interpreted in research or applied settings, however, the methodological robustness and reproducibility of the assessment procedures themselves must first be established.

The Institute for Neuro-Physiological Psychology (INPP) framework provides standardized observational procedures for assessing primitive reflex activity through predefined motor and postural responses elicited by specific testing positions and stimuli. Within this framework, reflex activity is typically rated using an ordinal scoring system, with higher scores representing greater observable expression of the reflex response. Although the INPP framework has been increasingly used in developmental and athletic populations [10,12,20,21,24,25,26], robust psychometric evidence supporting the reproducibility of its scoring procedures remains limited. Little is known regarding intra-rater consistency, inter-rater agreement, and the potential influence of evaluator expertise on scoring variability. This issue is methodologically important because the INPP framework relies on standardized observational ratings of motor and postural responses, including subtle distinctions between adjacent ordinal categories and qualitative interpretations of postural adjustments, balance alterations, and movement responses [26]. As with many observer-based clinical assessments, the reproducibility of the measurements may depend not only on the clarity of the scoring criteria but also on the evaluator’s experience, perceptual interpretation, and familiarity with the assessment procedures. Establishing the reproducibility of these assessments in young athletes is particularly relevant because previous findings have identified substantial inter-individual variability in PR activity within this population and suggested potential associations with sport-related performance. Before such differences can be meaningfully interpreted as reflecting individual sensorimotor characteristics, it is necessary to determine whether they can be distinguished from variability attributable to the assessment procedure or the evaluator. Consequently, establishing the reliability of PR assessments represents a necessary methodological step before their potential relevance in research or applied sport settings can be further investigated.

Therefore, the aim of the present study was to investigate the intra- and inter-rater reliability of subjective PR assessments performed using the INPP framework in elite youth football athletes and to examine whether evaluator expertise influenced scoring reproducibility by comparing experienced and novice raters. We hypothesized that the INPP assessment framework would demonstrate acceptable overall reproducibility, although some variability in reliability indices might emerge according to evaluator experience level.

## 2. Material and Methods

### 2.1. Participant Characteristics

Players were eligible for inclusion if they had completed a minimum of five years of structured training within a sports club prior to joining the academy and had remained injury-free for at least one month prior to the PR assessment. As the study was conducted within a fixed cohort, no a priori sample size calculation was used to determine recruitment. All 69 available players met the predefined eligibility criteria and were therefore included in the study. Written informed consent was obtained from all players and, when applicable, from their legal guardians prior to inclusion. The study was approved by the Ethics Committee (Comité de Protection des Personnes Sud Méditerranée 4; ID-RCB: 2019-A03012-55; approval date: 16 April 2020) and conducted in accordance with the Declaration of Helsinki, as well as local legislation and institutional requirements.

### 2.2. Study Design—Primitive Reflex Assessment

PR responses were assessed using a standardized protocol consistent with previous studies [20,21] and based on the INPP framework [26]. The assessed reflexes included the Moro reflex, asymmetrical tonic neck reflex (ATNR; left and right), symmetrical tonic neck reflex (STNR; flexion and extension), and tonic labyrinthine reflex (TLR; flexion and extension).

- The Moro reflex was assessed with participants standing upright, feet together, arms extended forward at shoulder height, eyes closed, wrists flexed, and hands relaxed, with the head slightly extended. Following standardized instructions and confirmation of readiness, participants performed a controlled backward fall into the examiner’s hands. Reflex activity was evaluated based on involuntary motor and postural responses, including arm abduction, loss of postural control, or signs of distress.
- The ATNR was assessed with participants standing, feet together, arms extended forward at shoulder height, eyes closed, wrists flexed, and hands relaxed. The examiner passively rotated the head to each side (left: ATNR L; right: ATNR R) using a slow and continuous movement followed by a 5–10 s pause at the end of each rotation and in the neutral position. Reflex activity was evaluated based on involuntary movements of the arms or hands in response to head rotation.
- The STNR was assessed in a quadruped position with hands under the shoulders and knees under the hips. Participants were instructed to maintain the quadruped position while the examiner induced head flexion (STNR FLX) and extension (STNR EXT), each followed by a 5 s pause. Reflex activity was evaluated based on observable involuntary responses, including tremors of the upper limbs, hip displacement, arm flexion, or trunk extension.
- The TLR was assessed with participants standing, feet together, and eyes closed. The test consisted of continuous head extension (TLR EXT) and flexion (TLR FLX), each followed by a 5 s pause. Reflex activity was evaluated based on observable alterations in postural control, including forward or backward sway and loss of balance.

Reflex responses were scored on an ordinal scale ranging from 0 (no observable response) to 4 (marked involuntary response), based on the magnitude of involuntary movement observed following each stimulus. Detailed scoring criteria for each reflex are provided in the Supplementary Material (Primitive Reflex Assessment and Scoring Criteria).

Intra-rater reliability was assessed by an experienced examiner across three testing sessions conducted at three-month intervals over a six-month period. The three-month interval was selected to minimize potential recall and familiarization effects between successive assessments and to examine the reproducibility of PR scoring over a longer time frame than conventional short-term test–retest protocols. All testing sessions were performed during designated rest days within the training week to standardize the immediate training context as far as possible.

Inter-rater reliability was evaluated using video recordings obtained during the initial testing session. Simultaneous live assessment by all three raters was not feasible because the evaluators were not available at the same time. The use of standardized video recordings therefore provided a practical solution while also ensuring that all evaluators scored the same elicited motor and postural responses, thereby minimizing variability related to repeated test administration and allowing the analysis to focus specifically on agreement in PR scoring. Recordings were standardized for camera placement, viewing angle, and testing conditions to ensure identical visual information across evaluators. Two additional novice raters independently scored all PR responses from the video recordings using the same INPP assessment procedures and scoring criteria.

A global score was calculated as the sum of individual reflex scores to quantify overall PR activity, as previously described [9]. Based on established criteria, the global score was used to categorize PR activation into five levels: <10% (null), ≥10% to <25% (low), ≥25% to <50% (medium), ≥50% to <75% (high), and ≥75% (maximal), corresponding to global score ranges of 0–2, 3–7, 8–13, 14–20, and 21–28, respectively [26,27].

Players were categorized as having inactive PRs when the global score was ≤2 or, at the individual level, when the score for a given reflex was 0. Conversely, players were classified as having active PRs when the global score was >2 or, at the individual level, when the score for a given reflex was >0. Thus, at the individual-reflex level, any observable involuntary response corresponding to a score of 1–4 was considered indicative of active reflex activity.

### 2.3. Statistical Analysis

Statistical analyses were performed using Jamovi (version 2.7.38). Descriptive statistics are reported as mean ± standard deviation. Mean differences (∆) were calculated for repeated measurements. Relative intra- and inter-rater reliability was assessed using intraclass correlation coefficients (ICCs) with corresponding 95% confidence intervals (95% CIs). ICCs were calculated using a two-way model with absolute agreement and single measures. ICC values were interpreted according to the recommendations of Koo and Li [28]: <0.50, *poor*; 0.50–0.75, *moderate*; 0.75–0.90, *good*; and >0.90, *excellent* reliability. Absolute reliability was quantified using the standard error of measurement (SEM) and the minimal detectable change at the 95% confidence level (MDC95). The SEM was calculated as the square root of the residual variance derived from the ICC model, and the MDC95 was calculated as $1.96\cdot \sqrt{2}\cdot$ SEM. Both SEM and MDC95 were expressed in the same units as the corresponding PR scores. The reporting of this reliability study followed the Guidelines for Reporting Reliability and Agreement Studies recommendations [29].

## 3. Results

### 3.1. Participant Characteristics

All 69 included players completed the assessments and were retained for analysis. Participants were male elite youth football players (age: 18 ± 2 years; height: 179 ± 6 cm; body mass: 70 ± 7 kg) and had been enrolled in the academy for an average of 2 ± 2 years. Playing positions were distributed as follows: 10 goalkeepers, 16 central defenders, 8 full backs, 18 midfielders, 12 wingers, and 5 forwards. Forty-seven players competed within their chronological age category (“non-upgraded”), whereas 22 competed in a higher-age category (“upgraded”).

### 3.2. Primitive Reflex Activity

Based on the global score, 81–86% of players were classified as having active PRs across assessments (Table 1). At the individual-reflex level, the prevalence of active responses ranged from 29% to 90%, depending on the reflex assessed, with broadly similar proportions observed across testing sessions and raters (Table 1).

### 3.3. Intra-Rater Reliability

The global score demonstrated *excellent* relative intra-rater reliability (ICC = 0.97; 95% CI [0.96–0.98]), with a SEM of 0.6 and an MDC95 of 1.7 points (Table 2). Across individual reflexes, relative reliability ranged from *good* to *excellent* (ICC = 0.86–0.98; 95% CI = [0.81–0.99]; Table 2). Absolute reliability indices showed SEM values ranging from 0.1 to 0.3 points and MDC95 values ranging from 0.3 to 0.9 points across individual reflexes. Mean differences between repeated assessments remained minimal for both the global score and individual reflexes (|∆| ≤ 0.1; Table 2).

### 3.4. Inter-Rater Reliability

The global score demonstrated *excellent* video-based inter-rater relative reliability (ICC = 0.96; 95% CI [0.95–0.98]), with a SEM of 0.7 and an MDC95 of 2.0 points (Table 3). Across individual reflexes, relative reliability ranged from *moderate* to *excellent* (ICC = 0.69–0.99; 95% CI [0.56–0.99]). SEM values ranged from 0.1 to 0.4 points and MDC95 values from 0.3 to 1.1 points across individual reflexes (Table 3). Mean differences between raters remained small for both the global score and individual reflexes (|∆| ≤ 0.3; Table 3).

### 3.5. Pairwise Inter-Rater Reliability

*Excellent* relative reliability was observed for the global score across all rater pairs including comparisons between the expert and novice raters (R1–R2 and R1–R3) and between the two novice raters (R2–R3), with ICC values ranging from 0.95 to 0.99 (Table 4). For the global score, SEM values ranged from 0.4 to 0.8 points and MDC95 values from 1.2 to 2.3 points. Across individual reflexes, relative reliability ranged from *moderate* to *excellent* (ICC = 0.57–0.99; 95% CI [0.35–0.99]), with SEM values ranging from 0.1 to 0.5 points and MDC95 values from 0.2 to 1.3 points (Table 4).

## 4. Discussion

The present study aimed to investigate the intra- and inter-rater reliability of subjective PR assessments performed using the INPP framework and to examine the influence of evaluator expertise by comparing expert and novice raters in youth football athletes. In agreement with our hypothesis, the INPP assessment framework demonstrated overall *good* to *excellent* intra-rater relative reliability and *moderate* to *excellent* video-based inter-rater relative reliability, together with generally low absolute measurement error as indicated by the SEM and MDC95 values. Agreement between the experienced examiner and novice raters also ranged from *moderate* to *excellent* across reflexes, while the global score consistently demonstrated *excellent* reliability. Overall, these findings suggest that evaluator experience had a limited influence on scoring reproducibility when standardized assessment procedures were applied, although reflex-specific differences in measurement error and agreement remained evident.

The *excellent* intra- and inter-rater reliability observed for the global score is particularly relevant from a psychometric perspective. The global score showed ICC values of 0.97 for intra-rater reliability and 0.96 for video-based inter-rater reliability, accompanied by SEM values of 0.6–0.7 points and MDC95 values of 1.7–2.0 points. Thus, the high relative reliability of the global score was also supported by relatively low absolute measurement error. Reliability is a prerequisite for both scientific inference and clinical interpretation [29,30], especially in observer-based assessments where measurement variability may arise from repeated evaluations by the same examiner or from evaluations performed by different raters [31,32]. Insufficient reliability increases measurement error, reduces interpretability, and limits the ability to distinguish true inter-individual differences from variability attributable to the assessment procedure itself [33]. Comparable levels of reliability have been reported for other standardized observer-based movement assessments. For example, Mann et al. [34] reported inter-rater ICCs of 0.73–0.85 among novice raters and 0.71–0.88 among expert raters for the composite score of a movement competency screen in youth athletes. Similarly, Udompanich et al. [35] reported *excellent* inter-rater reliability (ICC = 0.94–0.98) between experienced and novice raters across several clinical balance tests in young physically active individuals. Although these assessments differ conceptually from PR testing, they support the broader observation that standardized observational procedures can yield reproducible composite outcomes across evaluators with different levels of experience. Because the global score aggregates responses across multiple reflexes, it may reduce the influence of isolated scoring variability and provide a more stable estimate of overall PR activity [36,37]. This finding strengthens the interpretability of global PR activity levels, which previous studies using the INPP framework in developmental or athletic populations have generally examined without robust evidence regarding the reproducibility of the scoring procedures themselves [10,20,21].

Reliability indices nevertheless varied across individual reflexes for both intra- and inter-rater assessments, indicating that not all PR evaluations were equally reproducible. TLR EXT showed the lowest video-based inter-rater reliability (ICC = 0.69; SEM = 0.4; MDC95 = 1.1), whereas STNR FLX demonstrated the highest inter-rater reliability (ICC = 0.99; SEM = 0.1; MDC95 = 0.3). Pairwise analyses reinforced this contrast, with TLR EXT reaching an ICC as low as 0.57 and an MDC95 of 1.3 points for one expert–novice comparison. Given the restricted 0–4 scoring scale, an MDC95 approaching or exceeding one point suggests that relatively large score differences may be required to exceed measurement error for some individual reflexes. These differences may be explained by the nature of the scoring criteria. STNR assessments rely on relatively explicit motor or postural responses, thereby limiting interpretational ambiguity during scoring. In contrast, TLR assessment requires discrimination between more subtle gradations of postural instability and balance alteration, which may increase scoring variability between evaluations and raters. Similarly, the ATNR scoring criteria involve subtle distinctions between adjacent ordinal categories, potentially contributing to slightly lower reproducibility compared with STNR reflex assessments (see Supplementary Material—Primitive Reflex Assessment and Scoring Criteria). Such qualitative distinctions between adjacent ordinal categories are known to increase interpretational variability in observer-based scales [29,31,32]. A similar influence of scoring complexity has been reported for other observational movement assessments. In the Selective Functional Movement Assessment, Glaws et al. [38] reported intra-rater ICCs of 0.86, 0.71, and 0.59 for evaluators with decreasing levels of experience, respectively, while overall inter-rater reliability was only 0.43. These findings suggest that evaluator-dependent variability may become more pronounced when scoring requires discrimination of subtle or multidimensional movement features. In addition, STNR FLX responses were frequently scored as inactive in the present cohort (63%) compared to TLR EXT (14%), which may also have facilitated agreement between evaluators by reducing score variability. Overall, these findings suggest that reproducibility may partly depend on the qualitative complexity of the observable responses and on the precision of the associated scoring criteria.

The pairwise inter-rater analyses indicate that evaluator experience did not uniformly determine scoring reproducibility when standardized assessment procedures were applied. The global score demonstrated *excellent* reliability across all rater pairs (ICC = 0.95–0.99), including both expert–novice and novice–novice comparisons. However, greater between-pair variability was evident for individual reflexes, further suggesting that the influence of evaluator experience may depend on the complexity of the response being scored. This pattern is consistent with findings from other standardized movement assessments. Minick et al. [39] compared expert and novice raters using standardized video recordings of the Functional Movement Screen, which also relies on ordinal observational scoring. Expert–novice comparisons generally demonstrated *substantial* to *excellent* agreement across the assessed components. Similarly, Mann, O’Dwyer, Bruton, Bird and Edwards [34] reported broadly comparable reliability of movement competency composite scores between novice and expert raters, although reliability of individual movement scores was more variable. These findings suggest that evaluator experience alone does not necessarily determine observational reliability when procedures and scoring criteria are standardized. Standardized testing positions and externally induced stimuli likely contributed to reducing procedural ambiguity and limiting evaluator-dependent variability in the present study. In this context, novice raters were able to interpret involuntary responses consistently despite limited prior experience with PR assessments. Indeed, standardized scoring procedures and evaluator familiarization were previously shown to contribute to improving inter-rater agreement [29,30]. Nevertheless, the lower reliability and larger measurement error observed for some reflexes involving subtle postural alterations, particularly TLR EXT, indicate that evaluator experience and perceptual interpretation may still influence scoring when responses are more ambiguous or visually nuanced. Further studies should specifically examine the amount of training and familiarization required to optimize PR scoring reliability.

From an applied perspective, the present findings indicate that standardized INPP-based PR assessments can provide reproducible scores in elite youth football players, including when ratings are performed by evaluators with limited prior experience. This is relevant for both research and applied sport environments, where measurement procedures must demonstrate sufficient reproducibility before inter-individual differences can be meaningfully interpreted. In particular, the excellent relative reliability and relatively low absolute measurement error observed for the global score suggest that aggregated PR activity may provide a comparatively stable descriptive measure of individual sensorimotor characteristics. However, the present findings should not be interpreted as demonstrating that PR assessments are suitable for talent identification or clinical screening. Their potential practical relevance remains contingent on further evidence regarding construct validity, relationships with meaningful sport-performance outcomes, responsiveness to change, and practically meaningful interpretative thresholds. The present study should therefore primarily be considered as establishing an important methodological prerequisite for future investigations into the potential applied value of PR assessment in sport.

Several limitations should be considered when interpreting the present findings. First, the sample consisted exclusively of male youth football athletes from an elite academy, which may limit the generalizability of the results to female athletes, younger or older populations, and clinical groups. Second, no formal a priori sample size calculation was performed because all available players meeting the eligibility criteria within the academy cohort were included. Nevertheless, according to the precision-based approach proposed by Bonett [40], 68 participants are required, with three observations per participant, to estimate an ICC of 0.70 with a 95% confidence interval width of 0.20. Thus, although the sample size was not determined prospectively, the final sample of 69 players met this precision-based benchmark for the primary reliability analyses. Third, the three-month interval between repeated assessments was longer than that commonly used in short-term test–retest reliability studies. Although this interval minimized potential recall and familiarization effects, genuine changes in PR activity between sessions cannot be completely excluded. Consequently, the intra-rater estimates reported here should be interpreted as reflecting reproducibility over a three-month interval rather than short-term repeatability alone. Fourth, inter-rater assessments were conducted using standardized video recordings rather than simultaneous live evaluations. This approach was partly motivated by the fact that the three raters were not available at the same time and therefore could not perform concurrent live assessments. The use of video recordings also ensured that all raters evaluated identical motor and postural responses, thereby limiting variability related to test administration and focusing the analysis on scoring agreement. However, INPP assessments are typically administered and scored in real time, and video-based evaluation may not capture all sensory, spatial, and contextual information available during direct examination. Consequently, the present inter-rater estimates should primarily be interpreted as reflecting the reproducibility of PR scoring under standardized observational conditions rather than the full reproducibility of live test administration and scoring. Future studies should therefore examine inter-rater reliability during simultaneous live assessments. Fifth, the present study focused on four PRs commonly assessed within the INPP framework, and reliability may differ for other reflexes or assessment procedures. Future investigations should therefore examine additional PRs frequently described in neurological examination, such as the Babinski, Landau, or grasp reflexes [41]. Finally, the present study focused on reliability and measurement error and did not assess other measurement properties, such as construct validity or responsiveness. Although SEM and MDC95 quantify the expected measurement error and the magnitude of score differences required to exceed it, they do not indicate whether such differences are meaningful from a clinical or sport-performance perspective.

## 5. Conclusions

The present study showed that subjective INPP-based assessments of the Moro reflex, ATNR, STNR, and TLR exhibited *good* to *excellent* intra-rater relative reliability and *moderate* to *excellent* video-based inter-rater relative reliability in elite youth football athletes, together with generally low absolute measurement error as reflected by SEM and MDC95 values. Agreement remained *moderate* to *excellent* among experienced and novice raters, while the global score consistently demonstrated *excellent* reliability, suggesting that standardized assessment procedures may help limit variability associated with evaluator experience despite the observational nature of the scoring system. Overall, the present findings provide preliminary psychometric support for the reproducibility of INPP-based PR assessments and reinforce their potential applicability in research and applied sport settings.

## Figures and Tables

**Table 1:** Prevalence of active primitive reflexes according to rater and testing session.

Primitive Reflex	R1–T1	R1–T2	R1–T3	R2	R3
Global score	56 (81)	59 (86)	59 (86)	56 (81)	57 (83)
TLR EXT	61 (88)	62 (90)	61 (88)	57 (83)	56 (81)
TLR FLX	50 (72)	48 (70)	49 (71)	48 (70)	51 (74)
ATNR R	48 (70)	50 (72)	49 (71)	55 (80)	55 (80)
ATNR L	38 (55)	42 (61)	42 (61)	43 (62)	43 (62)
Moro reflex	21 (30)	20 (29)	20 (29)	20 (29)	22 (32)
STNR EXT	23 (33)	24 (35)	23 (33)	24 (35)	24 (35)
STNR FLX	25 (36)	26 (38)	25 (36)	26 (38)	26 (38)

Note. Values are presented as N (%), indicating the number and percentage of players classified as having active PRs. At the individual-reflex level, a primitive reflex was classified as active when its score was >0. At the global level, primitive reflex activity was classified as active when the global score was >2. R1: expert rater; R2 and R3: novice raters; T1: test 1; T2: test 2; T3: test 3; TLR: tonic labyrinthine reflex; ATNR: asymmetrical tonic neck reflex; STNR: symmetrical tonic neck reflex; EXT: extension; FLX: flexion; R: right; L: left.

**Table 2:** Descriptive statistics and intra-rater relative and absolute reliability for primitive reflex assessments (global score and individual reflexes) across the three testing sessions.

Primitive Reflex	Mean Score			∆ Score			Reliability		
	Test 1	Test 2	Test 3	T1–T2	T2–T3	T1–T3	ICC [95% CI]	SEM	MDC95
Global score	6.0 ± 3.9	6.0 ± 3.7	5.9 ± 3.6	0.0 ± 0.9	0.1 ± 0.5	0.1 ± 1.1	0.97 [0.96–0.98]	0.6	1.7
TLR EXT	1.4 ± 0.8	1.4 ± 0.8	1.3 ± 0.8	0.0 ± 0.4	0.1 ± 0.3	0.1 ± 0.5	0.86 [0.81–0.91]	0.3	0.8
TLR FLX	1.0 ± 0.8	1.0 ± 0.8	1.0 ± 0.8	0.0 ± 0.4	0.0 ± 0.2	0.0 ± 0.3	0.93 [0.90–0.96]	0.2	0.6
ATNR R	1.0 ± 0.9	1.0 ± 0.9	1.0 ± 0.9	0.0 ± 0.5	0.0 ± 0.2	0.0 ± 0.5	0.87 [0.82–0.92]	0.3	0.8
ATNR L	0.8 ± 0.8	0.8 ± 0.8	0.8 ± 0.8	−0.1 ± 0.3	0.0 ± 0.1	−0.1 ± 0.4	0.93 [0.90–0.96]	0.2	0.6
Moro reflex	0.9 ± 1.5	0.8 ± 1.4	0.9 ± 1.4	0.1 ± 0.6	0.0 ± 0.1	0.0 ± 0.6	0.95 [0.92–0.96]	0.3	0.9
STNR EXT	0.4 ± 0.7	0.5 ± 0.7	0.4 ± 0.7	0.0 ± 0.2	0.0 ± 0.2	0.0 ± 0.3	0.94 [0.91–0.96]	0.2	0.5
STNR FLX	0.5 ± 0.9	0.5 ± 0.9	0.5 ± 0.9	0.0 ± 0.2	0.0 ± 0.1	0.0 ± 0.2	0.98 [0.97–0.99]	0.1	0.3

Note. Values are presented as mean ± standard deviation. ∆: mean differences; T1: test 1; T2: test 2; T3: test 3; ICC: intraclass correlation coefficient; CI: confidence interval; SEM: standard error of measurement; MDC95: minimal detectable change at the 95% confidence level; TLR: tonic labyrinthine reflex; ATNR: asymmetrical tonic neck reflex; STNR: symmetrical tonic neck reflex; EXT: extension; FLX: flexion; R: right; L: left.

**Table 3:** Descriptive statistics and inter-rater relative and absolute reliability for primitive reflex assessments (global score and individual reflexes) across the three raters.

Primitive Reflex	Mean Score			∆ Score			Reliability		
	Rater 1	Rater 2	Rater 3	R2–R1	R3–R1	R2–R3	ICC [95% CI]	SEM	MDC95
Global score	6.0 ± 3.9	5.8 ± 3.7	5.9 ± 3.6	−0.2 ± 1.1	−0.1 ± 1.2	0.0 ± 0.6	0.96 [0.95–0.98]	0.7	2.0
TLR EXT	1.4 ± 0.8	1.1 ± 0.7	1.1 ± 0.7	−0.2 ± 0.6	−0.3 ± 0.7	0.1 ± 0.4	0.69 [0.56–0.79]	0.4	1.1
TLR FLX	1.0 ± 0.8	1.0 ± 0.8	1.0 ± 0.8	−0.1 ± 0.4	0.0 ± 0.5	0.0 ± 0.4	0.83 [0.76–0.88]	0.3	0.9
ATNR R	1.0 ± 0.8	1.1 ± 0.8	1.1 ± 0.8	0.1 ± 0.6	0.1 ± 0.5	0.0 ± 0.2	0.85 [0.78–0.90]	0.3	0.9
ATNR L	0.8 ± 0.8	0.9 ± 0.8	0.9 ± 0.8	0.1 ± 0.4	0.1 ± 0.4	0.0 ± 0.2	0.91 [0.86–0.94]	0.2	0.7
Moro reflex	0.9 ± 1.5	0.8 ± 1.4	0.9 ± 1.4	−0.1 ± 0.6	0.0 ± 0.6	−0.1 ± 0.3	0.93 [0.90–0.95]	0.4	1.0
STNR EXT	0.4 ± 0.7	0.5 ± 0.7	0.4 ± 0.7	0.0 ± 0.2	0.0 ± 0.3	0.0 ± 0.2	0.95 [0.93–0.97]	0.2	0.4
STNR FLX	0.5 ± 0.9	0.5 ± 0.9	0.5 ± 0.9	0.0 ± 0.2	0.0 ± 0.1	0.0 ± 0.1	0.99 [0.98–0.99]	0.1	0.3

Note. Values are presented as mean ± standard deviation. ∆: mean differences; R1: expert rater; R2: novice rater; R3: novice rater; ICC: intraclass correlation coefficient; CI: confidence interval; SEM: standard error of measurement; MDC95: minimal detectable change at the 95% confidence level; TLR: tonic labyrinthine reflex; ATNR: asymmetrical tonic neck reflex; STNR: symmetrical tonic neck reflex; EXT: extension; FLX: flexion; R: right; L: left.

**Table 4:** Pairwise inter-rater relative and absolute reliability for primitive reflex assessments (global score and individual reflexes) among the expert and novice raters.

Primitive Reflex	R1–R2 ICC [95% CI] SEM MDC95	R1–R3 ICC [95% CI] SEM MDC95	R2–R3 ICC [95% CI] SEM MDC95
Global score	0.96 [0.93–0.97] 0.8 2.2	0.95 [0.92–0.97] 0.8 2.3	0.99 [0.98–0.99] 0.4 1.2
TLR EXT	0.67 [0.48–0.80] 0.4 1.1	0.57 [0.35–0.72] 0.5 1.3	0.84 [0.75–0.90] 0.3 0.8
TLR FLX	0.87 [0.79–0.91] 0.3 0.8	0.77 [0.66–0.85] 0.4 1.0	0.85 [0.76–0.90] 0.3 0.9
ATNR R	0.76 [0.64–0.84] 0.4 1.1	0.82 [0.72–0.88] 0.4 1.0	0.98 [0.96–0.99] 0.1 0.3
ATNR L	0.86 [0.78–0.91] 0.3 0.8	0.89 [0.83–0.94] 0.3 0.7	0.97 [0.95–0.98] 0.1 0.4
Moro reflex	0.91 [0.86–0.95] 0.4 1.2	0.90 [0.84–0.94] 0.5 1.3	0.98 [0.96–0.99] 0.2 0.6
STNR EXT	0.96 [0.93–0.97] 0.1 0.4	0.92 [0.88–0.95] 0.2 0.5	0.97 [0.95–0.98] 0.1 0.3
STNR FLX	0.98 [0.97–0.99] 0.1 0.3	0.99 [0.99–0.99] 0.1 0.2	0.99 [0.99–0.99] 0.1 0.2

Note. R1: expert rater; R2: novice rater; R3: novice rater; ICC: intraclass correlation coefficient; CI: confidence interval; SEM: standard error of measurement; MDC95: minimal detectable change at the 95% confidence level; TLR: tonic labyrinthine reflex; ATNR: asymmetrical tonic neck reflex; STNR: symmetrical tonic neck reflex; EXT: extension; FLX: flexion; R: right; L: left.

## Data Availability

The raw data supporting the conclusions of this article will be made available by the authors on request.

## Supplementary Materials

The following supporting information can be downloaded at https://www.mdpi.com/article/10.3390/jfmk11030324/s1.

## Abbreviations

ATNR	asymmetrical tonic neck reflex
CI	confidence interval
EXT	extension
FLX	flexion
GS	global score
ICC	intraclass correlation coefficient
INPP	Institute for Neuro-Physiological Psychology
L	left
PR	primitive reflex
R	right
STNR	symmetrical tonic neck reflex
TLR	tonic labyrinthine reflex
