# Supplementary material for: Intra- and Inter-Rater Reliability of Primitive Reflex Assessments in Youth Football Athletes
Source: J Funct Morphol Kinesiol. 2026 Aug 21;11(3):324. doi: 10.3390/jfmk11030324 (PMC13510787; doi:10.3390/jfmk11030324)
Supplement: Supplementary file 1 [file jfmk-11-00324-s001.zip › jfmk-4428602-supplementary.pdf]

Supplementary Material: Primitive Reflex Assessment and Scoring Criteria

Primitive reflex	Test position	Protocol	Institute for Neuro-Physiological Psychology scoring scale
Tonic labyrinthine reflex Extension and flexion	Standing, feet together and eyes closed.	The test is performed under two conditions: first with the head in extension, and second with the head in flexion. In each condition, the subject must maintain their body position while moving the head between extension and flexion. It is important to observe any movements or compensations used to maintain balance and to ensure the subject's safety in case of loss of balance.	0. No response.<br>1. Slight alteration of balance and muscle tone because of change in head position.<br>2. Impairment of control of balance during test and/or alteration of muscle tone.<br>3. Near loss of balance and/or alteration of muscle tone and/or disorientation because of test procedure.<br>4. Loss of balance and/or marked adjustment in muscle tone in attempt to stabilize balance. This may be accompanied by dizziness or nausea.
Asymmetrical tonic neck reflex Right and left	Standing, feet together, arms extended perpendicular to the ground, wrists flexed, and hands relaxed.	The test consists of head rotation to both sides, induced by the examiner. The movement is slow and continuous, with a 5–10 second pause at each end of the rotation and in the neutral position. The sequence is repeated twice.	0. No movement of the arms in response to head rotation.<br>1. Slight deviation of the arm(s) up to 12–15° in the direction of head.<br>2. 30° rotation of the arms.<br>3. 45° rotation of the arms.<br>4. 90° rotation of the arms.
Moro reflex	The feet are together, with the arms extended forward, perpendicular to the ground, and the wrists relaxed. The eyes are closed, and the head is in extension.	After a clear explanation of the test and obtaining the subject's consent, the subject is instructed to perform a controlled backward lean, allowing the examiner to support them.	0. No movement of the arms and no sign of distress or discomfort.<br>1. Fractional movement of the arms outwards (abduction) or momentary freeze.<br>2. Definite arm involvement and slight freeze reaction and/or signs of emotional discomfort following test procedure.<br>3. Partial abduction of the arms and dislike of test procedure.<br>4. Abduction of the arms and/or visibly distressed by the test.
Symmetrical tonic neck reflex Extension	Quadruped position with hands under shoulders and knees under hips.	The instruction for the subject is to maintain a quadruped position while moving the head. Flex the head downward and hold this position for 5 seconds. Movement must be slow and continuous	0. No response.<br>1. Slight movement in the hips (flexion in the lower body).<br>2. Small movement in the hips.<br>3. Definite movement in the hips.<br>4. Movement of the bottom back onto the ankles so that the child is in the 'sitting cat' position.
Symmetrical tonic neck reflex Flexion	Quadruped position with hands under shoulders and knees under hips.	The instruction for the subject is to maintain a quadruped position while moving the head. Extend the head upward and hold this position for 5 seconds. Movement must be slow and continuous.	0. No response.<br>1. Tremor in one or both arms in response to flexion of the head.<br>2. Slight bending of the elbows.<br>3. Definite bending of the arms because of head flexion and/or elevation of the feet.<br>4. Arms collapse when the head is flexed.
